# Transition of Care in Young People With Attention-Deficit/Hyperactivity Disorder (ADHD) From Child to Adult Services

**DOI:** 10.1192/bjo.2024.485

**Published:** 2024-08-01

**Authors:** Sangwoo Richard Jung, Catherine Saunders, Rudolf Cardinal, Tamsin Ford

**Affiliations:** 1 Department of Psychiatry, University of Cambridge, Cambridge, United Kingdom; 2 GKT School of Medical Education, King's College London, London, United Kingdom

## Abstract

**Aims:**

This retrospective cohort study using routinely collected administrative clinical data from the Cambridgeshire and Peterborough Foundation Trust (CPFT) Research Database, aims to understand how many children and young people (CYP) with attention-deficit/hyperactivity disorder (ADHD) undergo successful transition from child and adolescent mental health services (CAMHS) and community paediatric services, to adult mental health services and investigate the factors that are associated with the successful transition of care in young people with ADHD to adult services.

Many young people with ADHD, in need of service transition from child to adult services, experience serious barriers in receiving the care they need, constrained by scarce resources, low capacity in specialist services and variable awareness or training across various levels of care.

**Methods:**

We explored the numbers and clinical and socio-demographic characteristics of CYP with ADHD who undergo successful transition from CAMHS and paediatric services, to adult mental health services. We will explore whether children with certain sociodemographic factors/treatment/service attended are more likely than others to successfully transition using multivariable logistic regression.

**Results:**

Note results are rounded for statistical disclosure control. We identified 24,240 unique CYP for whom a referral (age < 18) exists to CPFT between 1 Sep 2007 and 31 Aug 2019 (with follow up until 2020). Of this cohort, 2300 were referred at any time to any ADHD service, 1760 CYP had a record of ADHD medication in their clinical notes at any time of whom 1590 CYP had a record of ADHD medication under the age of 18. Of these 1590 CYP, 330 had at least 1 year follow up in the database before and after their 18^th^ birthday and a record of ADHD prescribing during the year before they turn 18. This is a cohort of CYP who should have transitioned from child to adult services. Of these 330, 160 (48%) had been referred to any ADHD service between their 17^th^ and 19^th^ birthday and 190 (58%) had any record of ADHD medication in the year after they turn 18. Further analyses will explore the characteristics of CYP who successfully transition, and we will carry out a series of sensitivity analyses.

**Conclusion:**

With an increase in the number of children with ADHD who are prescribed medication, we can expect an increasing cohort of emerging adults who need continued care. This study will provide evidence on the current state of care to help identify areas for improvement.

